# Rg1 Protects Hematopoietic Stem Cells from LiCl-Induced Oxidative Stress via Wnt Signaling Pathway

**DOI:** 10.1155/2022/2875583

**Published:** 2022-03-27

**Authors:** Ziling Wang, Jieyu Xia, Jing Li, Linbo Chen, Xiongbin Chen, Yanyan Zhang, Lu Wang, Yaping Wang

**Affiliations:** ^1^Laboratory of Stem Cells and Tissue Engineering, Basic Medical College, Chongqing Medical University, Chongqing 400016, China; ^2^Chongqing University Cancer Hospital and Chongqing Cancer Institute and Chongqing Cancer Hospital, Chongqing 400030, China; ^3^NHC Key Laboratory of Birth Defects and Reproductive Health (Chongqing Population and Family Planning Science and Technology Research Institute), Chongqing 400002, China; ^4^Department of Anatomy and Histology and Embryology, Basic Medical College, Chengdu University of Traditional Chinese Medicine, Chengdu, Sichuan 610075, China

## Abstract

**Background:**

Ginsenoside Rg1 is a major component of ginseng with antioxidative and antiaging effects, which is a traditional Chinese medicine. In this study, we investigated the potential spillover and mechanism of action of Rg1 on LiCl-driven hematopoietic stem cell aging.

**Results:**

Collect the purified Sca-1^+^ hematopoietic cells for differentiation ability detection and biochemical and molecular labeling. The experiment found that Rg1 plays an antiaging role in reversing the SA-*β*-gal staining associated with LiCl-induced hematopoietic stem cell senescence, the increase in p53 and p21 proteins, and sustained DNA damage. At the same time, Rg1 protects hematopoietic cells from the reduced differentiation ability caused by LiCl. In addition, Rg1 increased the excessive inhibition of intracellular GSK-3*β* protein, resulting in the maintenance of *β*-catenin protein levels in hematopoietic cells after LiCl treatment. Then, the target gene level of *β*-catenin can be maintained.

**Conclusions:**

Rg1 exerts the pharmacological effect of maintaining the activity of GSK-3*β* in Sca-1^+^ hematopoietic cells, enhances the antioxidant potential of cells, improves the redox homeostasis, and thus protects cells from the decline in differentiation ability caused by aging. This study provides a potential therapeutic strategy to reduce stem cell pool failure caused by chronic oxidative damage to hematopoietic stem cells.

## 1. Background

Adult stem cells have great potential for clinical application because of their multidirectional differentiation ability and easy acquisition. Hematopoietic stem cells (HSCs) are the most well-studied and most mature adult stem cell. Autologous and allogeneic hematopoietic stem cell transplantation (HSCT) has made remarkable progress in the treatment of malignant tumors of the blood system and other systems, autoimmune diseases, and genetic diseases, which has greatly promoted the treatment of these diseases, but morbidity and mortality associated with HSCT is still significant [1–4]. A common point in the pathogenesis of HSCT-related morbidity and mortality is the production of reactive oxygen species (ROS).

 ROS is formed as a natural byproduct of the normal metabolism of oxygen and plays an important role in cell signaling and homeostasis [5]. However, elevated ROS above the normal concentration will seriously damage the cell structure due to oxidative stress (OS) and DNA damage [6–9]. HSCs exposed to elevated ROS exhibit altered characteristics and undergo proliferation and differentiation after mobilization to oxygen-enriched blood flow, but this will cause the hematopoietic stem cell pool to be reduced/depleted [10–12]. Therefore, it is very important to understand the oxidative stress of hematopoietic stem cell aging.

Lithium chloride is commonly used to treat bipolar disorder. Research reports that LiCl treatment causes ROS accumulation to induce lipid accumulation [13]. LiCl can also inhibit the proliferation of primary schwannoma cells by transplanting the expression of apoptosis-related proteins [14]. However, the role of LiCl on hematopoietic stem cells has not been reported. In this study, LiCl was used to trigger oxidative stress to simulate the aging and damage environment of hematopoietic stem cells. Ginseng is a traditional Chinese medicine with antiaging, antioxidant, and anti-inflammatory effects. Ginsenosides play a major pharmacological role in various active ingredients of ginseng, among which Rg1 is the most active representative ingredient [15]. Previous literature reported that Rg1 has antiaging and antioxidative stress effects in multiple organs [16–19]. In this study, we found that Rg1 can relieve hematopoietic stem cell aging, inhibit oxidative stress, and protect hematopoietic stem cell differentiation. The mechanism may be related to regulating GSK-3*β* activity and regulating DNA damage.

## 2. Materials and Methods

### 2.1. Animals

C57BL/6 mice, 6–8 weeks old, were purchased from the Medical and Laboratory Animal Center of Chongqing (qualified number is SCXK yu (007-0001)) and housed in a temperature- and light-controlled room with free access to water and food. All experiments with the mice were performed in accordance with the institutional and national guidelines and regulations and approved by the Chongqing Medical University Animal Care and Use Committee.

### 2.2. Reagents

Ginsenoside Rg1 (purity >95%) was purchased from Hongjiu Biotech Co, Ltd. (Tonghua, China). Lithium chloride (purity >95%) was purchased from Tianjin Chemical Reagent Factory (Tianjin, China). IMDM medium, fetal bovine serum (FBS), and equine serum (ES) were purchased from Gibco (CA, USA). The anti-Sca-1^+^ Micro Bead kit was purchased from Miltenyi Biotech Co. (Bergisch Gladbach, Germany). The SA-*β*-gal Staining kit was purchased from Cell Signaling (Boston, USA). The CFU-mix culture media was purchased rom Stem Cell Co. (CA, USA). The ROS kits were purchased from Beyotime Biotechnology (Shanghai, China). The polyclonal rabbit anti-mouse P53, P21Cip1/Waf1 antibody, and goat anti-rabbit antibody were purchased from Proteintech (Chicago, America). The monoclonal rabbit anti-mouse *β*-catenin, GSK-3*β*, TCF-4, and *γ*-H2AX antibody were purchased from Cell Signaling Technology (Boston, America).

### 2.3. Isolation and Purification of Stem Cell Antigen 1 (Sca-1)^+^ HSC/HPCs from the Mouse Bone Marrow

After the mice were sacrificed by cervical dislocation, the femur and tibia were collected to obtain a suspension of bone marrow mononuclear cells. HSC/HPCs positive for Sca-1^+^ were isolated and purified by magnetic-activated cell sorting (MACS) [20]. The percentage of Sca-1^+^ HSC/HPCs was analyzed before and after the separation.

The Sca-1^+^ HSC/HPCs were divided into four groups: the control group: the cells were grown in IMDM medium (Waltham, MA, USA); Rg1-administration group (Rg1 group): the cells were treated with Rg1 (10^−2^ mmol/L) dissolved in IMDM; LiCl-administration group (LiCl group): the cells were treated with LiCl (10 mmol/L) dissolved in IMDM; and LiCl-administration plus Rg1 treatment group (LiCl + Rg1 group): the cells were treated with Rg1 (10^−2^ mmol/L) and LiCl (10 mmol/L) dissolved in IMDM. All the cells were grown in a humidified atmosphere at 37°C with 5% CO_2_. After 48 h, the Sca-1^+^ cell obtained were used for subsequent experimental measurement.

### 2.4. Mixed Colony-Forming Unit (CFU-Mix) of HSC/HPC Culture

Briefly, the cell concentration was adjusted to 1 × 10^4^/L, inoculated into a 96-well plate, 2 ml of CFU-Mix complete medium was added to each well, cultured in 5% CO_2_, and grown at 37°C for 7 days. The number of colony formations indicates the pluripotency of Sca-1^+^ HSC/HPC [20].

### 2.5. Senescence-Associated *β*-Galactosidase (SA-*β*-Gal) Cytochemical Staining

The cell concentration was adjusted to 1 × 10^5^, washed twice with PBS, fixed at room temperature for 10 mins, washed twice with PBS, and stained with staining solution for 12 h at 37°C. Each group was randomly analyzed for 400 cells, and the number of blue positive cells was counted. The percentage of SA-*β*-gal-positive cells = number of blue cells/total number of cells.

### 2.6. Immunofluorescence Staining

The cell concentration was adjusted to 1 × 10^4^, 20–30 *μ*L of cell suspension was applied to the slide, and allowed to dry. 4% paraformaldehyde was fixed at room temperature for 10 mins, TBS was washed twice, 10% goat serum was blocked at room temperature for 1 h, and *β*-catenin (1 : 100), GSK-3*β* (1 : 100), and *γ*-H2AX (1 : 100) antibody was kept at 4°C overnight. TBS was washed twice and Cy3-labeled goat anti-rabbit IgG (1 : 300) was incubated for 1 h at room temperature in the dark. DAPI counterstained nuclei. Imaging was performed by fluorescence microscopy (LSM510; Carl Zeiss, Jena, Germany).

### 2.7. Western Blotting Analysis

Total protein was extracted and the concentration was measured using the BCA program. The loading was 50 *μ*g. The sample was separated by SDS-PAGE and transferred to a PVDF membrane. Anti-*β*-catenin, GSK-3*β*, TCF-4, P53, P21, *β*-actin, and Histone 2A were incubated overnight at 4°C. The TBST was washed 3 times and the secondary antibody (diluted 1 : 5000 in TBST) incubated at room temperature for 2 h. The chemiluminescence detection system (Bio-Rad) detected the amount of protein. *β*-actin and histone 2A were used as internal controls for cytoplasmic and nuclear proteins, respectively.

### 2.8. Measurement of ROS Level

ROS levels were measured using a kit (Beyotime Biotechnology Research Institute, Shanghai, China) probe (DCFH-DA). The concentration of each group of cells was adjusted to 1 × 10^6^, and the 2′,7′-dichlorofluorescein diacetate (DCFH-DA) probe was incubated in a cell incubator for 20 mins. ROS levels were determined using flow cytometry and laser scanning confocal microscopy (LSM510, Carl Zeiss, Jena, Germany).

### 2.9. Oxidation Damage Mark Analysis

According to the instructions provided by the reagent, the oxidation and oxidation level of cell lysis samples were measured using a CAT assay kit, a SOD assay kit, and a MDA assay kit (Beyotime).

### 2.10. RNA Extraction and Real-Time Quantitative RT-PCR

Control group (*n* = 3), cells were untreated. Experimental groups (*n* = 3), cells treated with Rg1 or LiCl. RNA was extracted immediately with RNAiso Plus (Takara) after the cells were treated. After nucleic acid quantification (DEPC water adjust concentration to 1000 ng/*μ*l) and contamination assessment with NanoDrop One/One^c^ UV-Vis (Thermo Fisher Scientific), total RNA was extracted and reverse transcribed into cDNA using Takara's extraction kit and reverse transcription kit (42°C, 2 min; 37°C, 15 min; 85°C, 5 s). Real-time PCR was performed using the BIO-RAD Sequence Detection System (FX96) (Bio-Rad, Inc., Pleasanton, CA, USA). *β*-actin was used as the internal reference gene. The reaction system (10 *μ*l) includes SYBR® Premix Taq™II (2×) (5 *μ*l), forward primer (0.1 *μ*l), reverse primer (0.1 *μ*l); cDNA (2 *μ*l), and RNase-free dH_2_O (2.8 *μ*l). The reaction conditions are 95°C 30 s; 95°C 5 s, 60°C 30 s, and circle 40 times. The 2^−ΔΔ Cq^ method was used to determine the relative level of mRNA. ΔCq = Cq (destination gene) − Cq (interior gene) and ΔΔCq = ΔCq (sample, Rg1 group, LiCl group, and Rg1 + LiCl group) − ΔCq (sample and control group). Each group was set up with 3 multiple holes and repeated 3 times. The PCR primers used are provided in the supporting information (Shanghai Freight Biological Engineering Co., Ltd) (Table 1).

### 2.11. Statistical Analyses

The SPSS 19.0 software (SPSS Inc., Chicago, IL, USA) was used for statistical analysis. The data are represented by the mean ± SD. Comparisons were made using one-way ANOVA and LSD tests. A difference of *P* < 0.05 was considered significant. All experiments were independent of three times.

## 3. Results

### 3.1. The Purity of Sca-1^+^ HSC/HPCs

**Figure 1:**
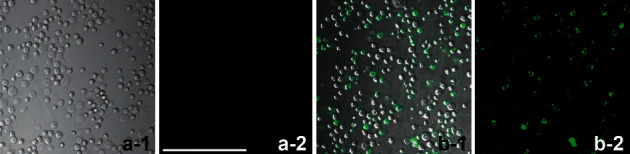
Expression of Sca-1^+^ in separated and purified cells. By further using adhesion magnetic beads purification, 83.32% of Sca-1^+^ HSC/HPCs was also obtained, which was 74.15% higher than the purity without purification. (a-1, b-1) merged picture of light microscope and Sca-1^+^ (green) fluorescent staining. (a-2, b-2) Sca-1^+^ (green) fluorescent staining. (a) Without purification. (b) Purified cells, Bar = 50 *μ*m.

The extracted bone marrow mononuclear cells were purified using MAC. The purified cells were detected for their surface antigen marker Sca-1 using flow cytometry, and cell viability was detected using the trypan blue exclusion method. The results showed that the proportion of Sca-1^+^ HSC/HPCs increased from 9.17% ± 1.06% to 83.32% ± 2.57% (Figure 1), and the cell survival rate did not change significantly to 98.2% ± 1.4%. It is suggested that the MAC method can separate and purify hematopoietic stem cells with good activity and purity, which lays the foundation for the next experiment.

### 3.2. Rg1 Protected Cells against LiCl-Induced Oxidative Stress

**Figure 2:**
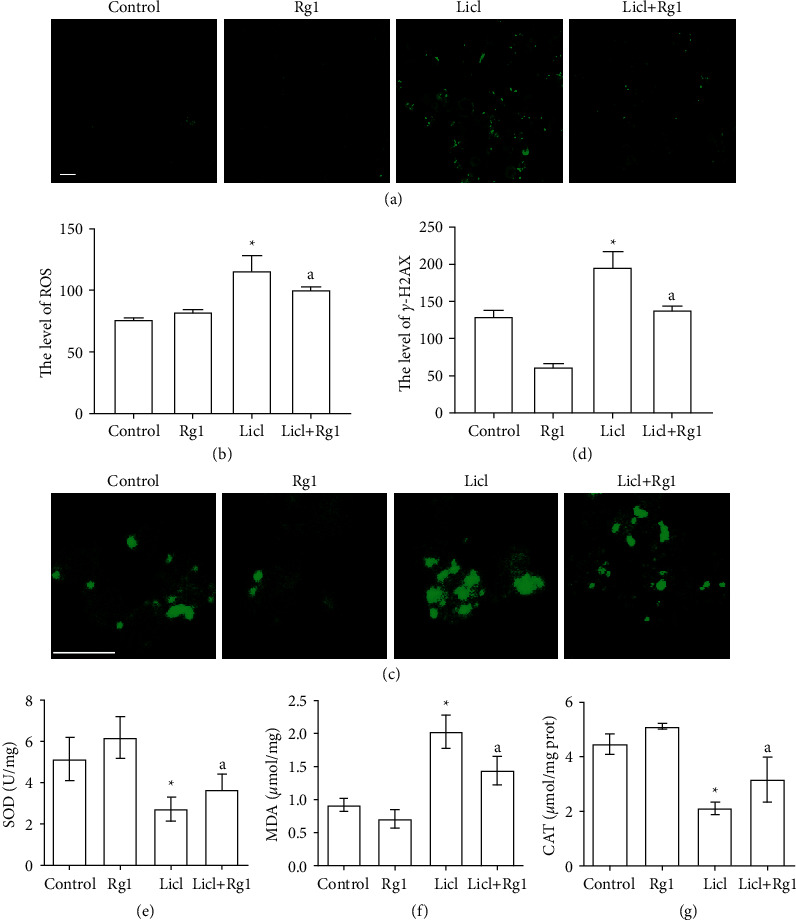
Effect of Rg1 on oxidative stress and nuclear damage in Sca-1^+^ HSC/HPCs. (a, b) The production of ROS by laser scanning confocal microscope assess, Bar = 10 *μ*m. (c, d) The expression and location of *γ*-H2AX which is an important marker for the formation of DNA damage foci; green fluorescence areas show the expression and location of *γ*-H2AX, Bar = 10 *μ*m. (e–g) The content of SOD, MDA, and CAT.  ^*∗*^*P* < 0.05 as compared with control group; ^a^*P* < 0.05 as compared with the LiCl group.

High levels of ROS can cause loss of HSC functionality. With LiCl process, the fluorescence of DCFH ROS increased significantly, and Rg1 attenuated the increase of ROS levels (Figures 2(a) and 2(b)). The expression of various antioxidant stress kinases can reflect the intracellular ROS damage; therefore, the activity of superoxide dismutase (SOD), catalase (CAT), and the content of malondialdehyde (MDA) were measured. Compared with the control group, LiCl significantly reduced the activities of antioxidant enzymes SOD and CAT and increased the content of MDA; however, Rg1 treatment improves the activity of antioxidant enzymes and reduces lipid oxidation products compared with the LiCl group (Figures 2(e)–2(g)).

The nuclear damage situation was further analyzed. The nuclear damage marker *γ*-H2A.X was used to evaluate the nuclear damage of hematopoietic stem cells. Compared with the control group, the expression of *γ*-H2A.X was increased in the LiCl group. After Rg1 treatment, the expression level of *γ*-H2A.X was significantly lower than that of the LiCl group (Figures 2(c) and 2(d)). In summary, the oxidative stress induced by LiCl in hematopoietic stem cells caused nuclear damage, while Rg1 treatment improved the damage.

### 3.3. Rg1 Protected Cells against LiCl-Induced Senescence

**Figure 3:**
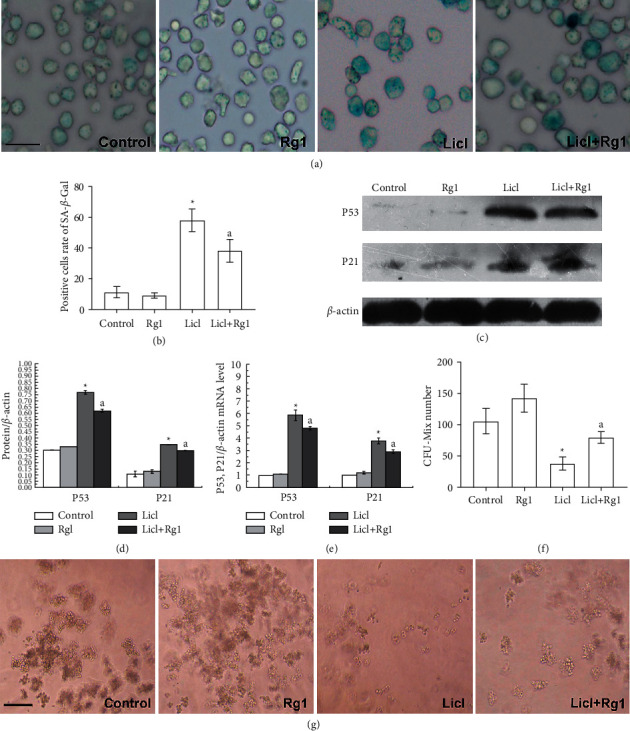
Effect of Rg1 on the differentiation and senescence in Sca-1^+^ HSC/HPCs. (a, b) SA-*β*-gal reflects the level of cell senescence; blue positive cells could be found in aging cells, Bar = 20 *μ*m. (c, d) The expression of P53 and P21 by western blot. (e) The mRNA level of p53 and p21. (f, g) Colony-forming capacity can react with the multidirectional differentiation properties of HSCs, Bar = 150 *μ*m.  ^*∗*^*P* < 0.05 as compared with the control group; ^a^*P* < 0.05 as compared with the LiCl group.

The condition of cell senescence can be detected by SA-*β*-gal staining, and positive cells are stained blue. Figures 3(a) and 3(b) shows that the number of SA-*β*-Gal-positive cells in the LiCl-treated group is large, and the staining is deep. After Rg1 treatment, the number of positive cells in the Rg1 + LiCl group decreased and the staining became lighter. p53 and p21^Cip1/Waf1^ are important markers of cellular senescence damage. As shown in Figures 3(c) and 3(d), p53 and p21^Cip1/Waf1^ protein expression was significantly enhanced in the LiCl group, while the LiCl + Rg1 group was lower than the LiCl group. The situation of mRNA expression is the same as that of protein expression (Figure 3(e)). These results indicated that nuclear damage caused by LiCl leads to aging changes in hematopoietic stems cells, and Rg1 protects cells from damage.

Notably, it is demonstrated for hematopoietic stems cells that aging contributes to impaired differentiation ability. Colony-forming capacity can react with the multidirectional differentiation properties of HSCs; as the HSCs age, the capacity to form CFU-Mix is gradually reduced. Therefore, we tested the ability of HSCs to form CFU-Mix. As shown in Figures 3(f) and 3(g), compared with that of the control group, there were much fewer CFU-Mix colonies and much fewer cells in each colony in the LiCl group. However, in the LiCl + Rg1 group, the number of CFU-Mix colonies was increased compared to the LiCl group.

### 3.4. Rg1 Enhances GSK-3*β* Activity and Reduces Wnt Signaling Pathway Expression

**Figure 4:**
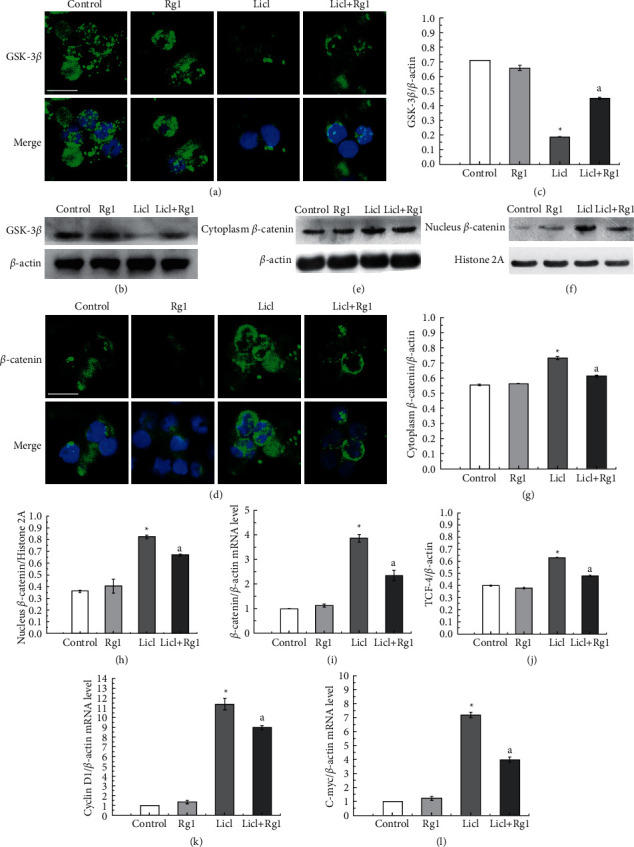
Effect of Rg1 on the expression and location of *β*-catenin, GSK-3*β*, and c-myc, and cyclin D1 mRNA and TCF-4 expression in Sca-1^+^ HSC/HPCs. ^*∗*^*P* < 0.05 as compared with the control group; ^a^*P* < 0.05 as compared with the LiCl group. Bar = 10 *μ*m.

In order to clarify the problems associated with GSK-3*β* in LiCl-induced hematopoietic stem cell oxidative damage and Rg1 protection, we evaluated the protein level of GSK-3*β*. Immunoblotting (Figures 4(b) and 4(c)) and immunofluorescence (Figure 4(a) showed that the expression of GSK-3*β* was significantly decreased in the LiCl group. The Wnt pathway is very important and highly conserved in the regulation of stem cell growth. Subsequently, *β*-catenin was found to increase in the LiCl group (Figures 4(d)–4(i)). Followed that decrease in *β*-catenin targeted genes TCF-4, cyclin D1 and c-myc were found in the LiCl group whereas elevation of TCF-4, cyclin D1, and c-myc was demonstrated in the LiCl + Rg1 group (Figures 4(j)–4(l)). These results indicate that the Wnt pathway may be involved in D-galactose LiCl-induced oxidative stress, nuclear damage, and hematopoietic stem cell aging. Rg1 plays a role in *β*-catenin upregulation to enhance cellular antioxidative potential meanwhile to alleviate lipid peroxidation, and to keep metabolic homeostasis, also to stabilize cell differentiation ability which may be the mechanism for protection of hematopoietic stem cells from aging and exhaustion of the stem cell pool.

## 4. Discussion

Hematopoietic stem cells (HSCs) are adult stem cells (ASCs) with multidirectional differentiation and self-renewal [21–23]. Hematopoietic stem cells can be isolated based on different physical properties (size and density), biochemical properties (enzymatic) [24], and surface antigen profiles [25, 26]. Sca-1 is widely regarded as a marker of mouse HSC and can be expressed on pluripotent HSCs. In this experiment, immunomagnetic bead sorting [27] was used to isolate hematopoietic cells positive for surface antigen Sca-1. The following flow cytometry was used to identify the purified cells. The successful sorting of hematopoietic stem cells has laid the foundation for subsequent research in cell biology.

HSC is very sensitive to the redox state of cells, so, the bone marrow cell niches where HSCs are present are hypoxic [28]. ROS refers to O_2_-free radicals and non-free radical derivatives that are produced during normal physiological processes which can regulate stem cell fate [29–31]. Furthermore, ROS can also directly modify metabolic enzymes [32] and various proteins [33] to participate in the regulation of stem cell metabolism. In this experiment, the intracellular ROS level of LiCl-treated cells was significantly increased, and Rg1 reversed this increase. In addition, ROS can attack DNA and lead to DNA double-stranded breakage (DBS) which activates ataxia telangiectasia mutated (ATM) [34, 35] and DNA-PKcs [36, 37] to form gamma-H2AX. Therefore, we measured the expression of H2AX protein to evaluate the effect of ROS on hematopoietic stem cells. The results showed that Rg1 alleviated the increased expression of *γ*-H2AX in hematopoietic stem cells caused by LiCl.

Otherwise, oxidative stress is triggered by an imbalance between ROS production and antioxidant defense. The “aging theory,” especially the “oxidative inflammatory aging hypothesis”, is closely related to oxidative stress. Oxidative stress biomarkers can be used as diagnostic tools or treatment goals. Cells contain multiple types of ROS scavengers (antioxidant enzymes) [38–40], which help prevent the excessive accumulation of ROS and repair oxidative damage to cells. Therefore, we detected superoxide dismutase (SOD) [41], catalase (CAT) [42], and glutathione peroxidase (GSH-px) [43]. The results suggested that Rg1 could enhance the activity of antioxidant enzyme and improve the ROS scavenging ability caused by LiCl. These preliminary results suggest Rg1 can resist oxidative damage by reducing cellular ROS levels and increasing antioxidant enzyme expression. Aging research and ROS have been closely linked since Harman (1972). The studies [44, 45] found that antioxidants increase cell proliferation and reduce cell senescence by reducing ROS, decreasing DNA damage, and reducing p16/Rb and p53/p21 signaling pathway. In addition, P21 and Akt regulate cell cycle arrest and ROS levels in aging-fibroblasts [46, 47]. Here, it was found that the p53 and p21 proteins in hematopoietic stem cells were significantly higher in the hematopoietic stem cells after LiCl treatment. Rg1 treatment reverses the increased protein expression caused by aging.

Aging has a variety of adverse effects on the hematopoietic system. Studies have shown that the anemia [48], immune system disorders [49], myelodysplastic syndrome (MDS) [50], and myelodysplastic disease (MPD) [51] are closely related to age. Previous studies have confirmed that ginsenoside Rg1 plays a role in antiaging [52], antioxidant, immune improvement, and neuronal growth through different targeted pathways. Ginsenoside Rg1 improves hepatic gluconeogenesis [53], promotes cerebral blood vessel formation in ischemic mice, and relieves cognitive impairment in aging mice through Akt signaling [54]. The Nrf2 signaling pathway [55], mTOR signaling pathway [56], TGF-*β*1 signaling pathway [57], and NF-*κ*B signaling pathway [56] may be interfered by Rg1, and target myocardial cells, podocytes, thereby preventing renal fibrosis [55], nerve ischemia perfusion injury [56], and lung fibrosis [57]. As regard as hematopoietic stem cells, research studies spotlighted on protective effects of Rg1 via reduction in oxidative stress and regulation of SIRT6 signaling pathway [58] and PI3K pathway [59]. In this experiment, we focussed on the Wnt pathway. The Wnt signaling pathway is highly conserved in evolution and regulates the maintenance and differentiation of stem cells. The results show that Rg1 protects hematopoietic stem cells from LiCl-induced oxidative stress may be related to the Wnt pathway, but considerably more work will need to be done to determine the target of Rg1. This is a worthy in-depth study, and we will consider it in the next research direction. Here, this study shows that it is now a realistic possibility to eliminate senescence of hematopoietic stem cells and restore cell differentiation through pharmacology. Ginsenoside Rg1 protects the effects of senescence of hematopoietic stem cells and reduces oxidative damage in the cells. Most importantly, this study provides new ideas for the clinical application of ginsenoside Rg1 to assist in the treatment potential of hematopoietic stem cells.

## Figures and Tables

**Table 1:** Primers used in real-time quantitative PCR.

p53	Forward	5′-CACGTACTCTCCTCCCCTCAA-3′
	Reverse	5′-GGCTCATAAGGTACCACCACG-3′

p21	Forward	5′-ATTCCTGGTGATGTCCGACC-3′
	Reverse	5′- AAAGTTCCACCGTTCTCGG-3′

*β*-catenin	Forward	5′-CAAGAAGCGGCTTTCAGTCG-3′
	Reverse	5′-CAGATCAGGCAGCCCATCAA-3′

c-myc	Forward	5′-AGGTGTGATATCCGGTAGA-3′
	Reverse	5′-CCTTCTAAGTGGTTGGAACA-3′

cyclin D1	Forward	5′-AGCTCCTGTGCTGCGAAGTGGAAAC-3′
	Reverse	5′-AGTGTTCAATGAAATCGTGCGGGG-3′

*β*-actin	Forward	5′-GCTACAGCTTCACCACCACAG-3′
	Reverse	5′-GGTCTTTACGGATGTCAACGTC-3′

## Data Availability

No data were used to support this study.
